# Early diagnosis of tuberous sclerosis complex: a race against time. How to make the diagnosis before seizures?

**DOI:** 10.1186/s13023-018-0764-z

**Published:** 2018-01-29

**Authors:** Monika Słowińska, Sergiusz Jóźwiak, Angela Peron, Julita Borkowska, Dariusz Chmielewski, Krzysztof Sadowski, Elżbieta Jurkiewicz, Aglaia Vignoli, Francesca La Briola, Maria Paola Canevini, Katarzyna Kotulska-Jóźwiak

**Affiliations:** 1 Department of Neurology and Epileptology, The Children’s Memorial Health Institute, Al. Dzieci Polskich 20, 04-730 Warszawa, Poland; 2 Department of Child Neurology, Medical University of Warsaw, Ul. Żwirki I Wigury 63A, 02-091 Warszawa, Poland; 3 Child Neuropsychiatry Unit - Epilepsy Center, San Paolo Hospital, Via Antonio di Rudinì, 8, 20142 Milan, Italy; 4 Department of Health Sciences, Università degli Studi di Milano, Via Antonio di Rudinì, 8, 20142 Milan, Italy; 5 Department of Pediatrics, Division of Medical Genetics, University of Utah School of Medicine, Salt Lake City, UT USA; 6 Department of Radiology, The Children’s Memorial Health Institute, Al. Dzieci Polskich 20, 04-730 Warszawa, Poland

**Keywords:** Tuberous sclerosis complex, TSC - early diagnosis, Infancy - cardiac tumors, Cardiac rhabdomyoma, Epilepsy, Preventative treatment, Antiepileptogenic treatment

## Abstract

**Background:**

Tuberous sclerosis complex (TSC) is a genetic disorder with an incidence of 1:6000 live births and associated with the development of benign tumors in several organs. It is also characterized by high rates of neurological and neuropsychiatric abnormalities, including epilepsy affecting 70–90% of patients and being one of the major risk factors of intellectual disability. The first seizures in TSC patients appear usually between the 4th and the 6th months of life. Recent studies have shown the beneficial role of preventative antiepileptic treatment in TSC patients, with the possibility for improvement of cognitive outcome. Moreover, European recommendations suggest early introduction of Vigabatrin if ictal discharges occur on EEG recordings, with or without clinical manifestation.

The aim of this study was to define the most useful approach to make the diagnosis of TSC before seizure onset (before age 4th months), in order to start early EEG monitoring with possible preventative treatment intervention.

**Methods:**

We performed a retrospective review of children who were suspected of having TSC due to single or multiple cardiac tumors as the first sign of the disease. We analyzed the medical records in terms of conducted clinical tests and TSC signs, which were observed until the end of the 4th month of age. Subsequently, we described the different clinical scenarios and recommendations for early diagnosis.

**Results:**

82/100 children were diagnosed with TSC within the first 4 months of life. Apart from cardiac tumors, the most frequently observed early TSC signs were subependymal nodules (71/100, 71%), cortical dysplasia (66/100, 66%), and hypomelanotic macules (35/100, 35%). The most useful clinical studies for early TSC diagnosis were brain magnetic resonance imaging (MRI), skin examination and echocardiography. Genetic testing was performed in 49/100 of the patients, but the results were obtained within the first 4 months of life in only 3 children.

**Conclusions:**

Early diagnosis of TSC, before seizure onset, is feasible and it is becoming pivotal for epilepsy management and improvement of cognitive outcome. Early TSC diagnosis is mostly based on clinical signs. Brain MRI, echocardiography, skin examination and genetic testing should be performed early in every patient suspected of having TSC.

## Background

Tuberous sclerosis complex (TSC) is a multisystem genetic disorder associated with the development of benign tumors (hamartomas) in several organs, including heart, brain, skin, kidney, liver and lungs. The prevalence of TSC is estimated as 1:6000 live births [1]. The disease is caused by heterozygous mutations in either *TSC1*, located on chromosome 9q34 and encoding hamartin, or *TSC2* located on chromosome 16p13.3 and encoding tuberin [1, 2]. Hamartin and tuberin form a heterodimer that suppresses the mTOR pathway, which coordinates various aspects of cell functioning, including cell growth, metabolism and proliferation [1]. Overactivation of the mTOR pathway in TSC patients is responsible for the development of tumors in different organs [1, 2]. TSC is the result of a de novo mutation in 2/3 of the affected people, while is inherited in 1/3 of the patients [1]. *TSC2* mutations are more frequent in sporadic cases and are usually associated with more severe clinical outcome [1, 2].

In TSC patients hamartomas may develop in various organs causing different symptoms [1–3]. Moreover, the clinical presentation differs between pediatric and adult individuals [3]. Whereas some signs may be observed even prenatally using antenatal ultrasonography or magnetic resonance imaging (MRI), other manifestations appear with age and are specific for the adult age [1, 3, 4]. Furthermore, disease severity may vary greatly from mild to severe in patients of the same age, even among family members [1]. Although the diagnosis of TSC is based mostly on clinical criteria, the recommendations from the Tuberous Sclerosis Consensus Conference in 2012 accepted the identification of a pathogenic mutation in either gene as sufficient to establish the diagnosis [4].

TSC is characterized by high rates of neurological and neuropsychiatric abnormalities, which represent a major cause of morbidity [5]. The vast majority of patients show central nervous system (CNS) manifestations including epilepsy, cognitive impairment and autism spectrum disorders [1, 5]. Epilepsy affects 70% to 90% of patients and is one of the most devastating comorbidities [1, 3, 6, 7]. Usually the first clinical seizures appear between the 4th and the 6th month of life [6–10]. However, the ictal abnormalities on EEG recordings predate clinical seizures [8, 10–13]. Moreover, the epileptogenic process begins even earlier during the latent period with alterations in gene expression, ion channel functions and synapses transmission [11]. Soon, paroxysmal discharges on EEG recordings occur, preceding clinical manifestations, lasting longer and longer, changing morphology, and finally leading to subtle partial seizures [8, 12]. These events may be easily overlooked by parents, and seizure onset may be noticed only late, with the beginning of generalized seizures [14].

Different types of seizures may be present and coexist in the course of TSC. The most destructive are infantile spasms, which occur in approximately 30–60% of patients [6, 8, 15]. It is documented that epilepsy, especially infantile spasms, is one of the major risk factors of cognitive impairment in TSC [6, 9, 10, 16, 17]. Usually children with earlier seizure onset present more severe developmental delay [16–18]. In the prospective study of Capal et al. on TSC patients the group of children without epilepsy developed better and had lower risk of autism spectrum behaviors compared to the group who suffered from seizures [17]. Moreover, earlier onset and higher seizure frequency, especially before 12 months of age, were also associated with poorer developmental outcome and increased risk of autism spectrum disorder [17]. Overall, the prevalence of cognitive impairment in TSC is about 40–70%, and more than 1/3 of the patients are profoundly impaired [6–10]. However, in the light of recent studies this prognosis may be improved.

Recent reports showed the beneficial role of early antiepileptic, or rather antiepileptogenic, intervention in TSC patients and the possibility for improvement of cognitive outcome [9, 10]. In our previous study, we treated with Vigabatrin a group of infants showing paroxysmal activity on EEG before the onset of clinical seizures (preventative treatment): that group achieved better seizure control and had better cognitive outcome at age 24 months compared to the group who was treated after clinical seizure onset [10]. Moreover, the significant number of preventively treated children did not develop epilepsy, and their EEG recordings - which were pathological at the beginning - normalized at the end of the study. The comparative, randomized study of both therapeutic approaches is currently continuing within the EPISTOP project [19]. Recent European recommendations on epilepsy treatment in TSC also suggest the early introduction of Vigabatrin treatment within 24 months of life if ictal discharges occur on EEG recordings, with or without clinical manifestation [18].

In the light of the recent studies and recommendations, it is therefore essential to make a diagnosis of TSC as soon as possible, in order to allow epileptogenic treatment before seizure onset, with a view to improvement of clinical outcome.

In this study, we propose the most useful approach to make the diagnosis of TSC in the newborn period and early infancy, before age 4 months, which is regarded as the usual time of clinical seizure onset.

## Methods

We performed a retrospective chart review of children born between 1990 and 2016 who were referred to the Department of Neurology and Epileptology of The Children’s Memorial Health Institute of Warsaw (68 children, which represents 20% of TSC population in our centre born between 1990 and 2016) and to the Child Neuropsychiatry Unit and Epilepsy Center of the San Paolo Hospital in Milan (32 children, which represents 13% of TSC population in our centre born between 1990 and 2016) with suspicion of TSC due to single or multiple cardiac tumors.

The study group was divided into three groups: patients with ‘early diagnosis of TSC’, established until the end of 16 weeks of age (4 months); patients with ‘late diagnosis of TSC’, made after 16 weeks of age; and patients with ‘possible TSC’, in whom the diagnosis was not certain.

We analyzed the medical records in terms of clinical tests that were conducted and TSC diagnostic signs that were observed within age 16 weeks. We applied the diagnostic criteria according to the recent recommendations from the Tuberous Sclerosis Consensus Conference in 2012 [3]. The diagnosis of TSC was confirmed in children with at least two major criteria or one major and two minor, or when a pathogenic mutation in either *TSC1* or *TSC2* was identified.

We reported the results of the clinical studies performed in our patients within the first 16 weeks of life. The utility of each particular test in early detection of TSC was defined as the percentage of studies conducted in the first 16 weeks of age that demonstrated diagnostic lesions.

Finally, we divided the group with ‘early TSC diagnosis’ into different clinical scenarios with the association of TSC diagnostic signs observed within the first 16 weeks of age.

The statistical analysis was performed with the STATISTICA 12 software using the Fisher exact test. Values of *P* < 0.05 were considered statistically significant.

## Results

A group of 100 patients was referred to our clinics due to single (14% 14/100) or multiple (86%, 86/100) cardiac tumors (rhabdomyomas) as the first possible presentation of TSC**.** The diagnosis of TSC was not confirmed in 5 patients out of 100 (5.0%) either by clinical studies or by molecular testing (the group of ‘possible TSC’). In 13 cases (13/100, 13.0%) the diagnosis of TSC was made after age 16 weeks (the group of ‘late TSC diagnosis’). Eighty-two patients out of 100 (82.0%) - 42 boys (51.2%) and 40 girls (48.8%) - were defined as the group with ‘early diagnosis of TSC’, as the disease had been confirmed before the end of the 16th week of age.

### Timing of TSC diagnosis

Cardiac tumors were detected prenatally in 71 individuals (71/100, 71.0%). Subsequently, antenatal brain MRI was conducted in 30 children (30/71, 42.3%) and prenatal diagnosis of TSC was confirmed in 20 patients (20/100, 20.0%) due to pathological lesions (cardiac tumors and subependymal nodules, cortical tubers or subependymal giant cell astrocytoma). Postnatal diagnosis of TSC was obtained in 75 patients (75/100, 75.0%). The age of postnatal diagnosis ranged from 1 day to 9.4 years (average age: 23.2 ± 67.6 weeks, median age: 4.5 weeks). The age of postnatal diagnosis ranged from 1 day to 16 weeks (average age: 5.3 ± 4.7 weeks, median age: 3.1 weeks) in the patients with ‘early’ diagnosis, and from 18 weeks to 9.4 years (average age: 2.1 ± 2.6 years, median age: 1.3 years) in the patients with ‘late’ diagnosis.

### Clinical tests

The clinical tests performed before the end of the 16th week of age were: antenatal or postnatal brain MRI, transfontanelle ultrasonography (TUS), skin examination (SE) including examination with Wood’s lamp, abdominal ultrasonography (US) or MRI, ophthalmological examination (OE) with fundoscopic exam, echocardiography, and genetic testing. Table 1 shows the results of the clinical tests conducted in the study group within the first 16 weeks of age, the average age of the first and last examination, and the age when pathological lesions were detected.

**Table 1:** Clinical tests performed prenatally or during the first 16 weeks of age in our cohort

Clinical studies		antenatal MRI	TUS	Brain MRI	SE	abdominal MRI	abdominal US	OE	PE	Genetic test
No. of tests conducted in the first 16 weeks showing diagnostic signs of TSC / no. of patients in whom the test was conducted within the first 16 weeks of age		20/30	18/54	76/83	35/73	2/7	8/63	7/33	98/98	3/3
		66.7%	33.3%	91.6%	47.9%	28.6%	12.7%	21.2%	100%	100%
Type of pathological lesions found in the first 16 weeks; no. of patients (% of positive results)		SEN	SEN	SEN	HMs	multiple renal cysts	multiple renal cysts	multiple retinal hamartomas	single cardiac tumors	*TSC1* mutation
		16/20	17/18	71/76	35/35	2/2	8/8	5/7	14/98	2/3
		80.0%	94.4%	93.4%	100.0%	100.0%	100.0%	71.4%	14.3%	66.7%
		cortical dysplasia	cortical dysplasia	cortical dysplasia	–	–	–	Achromic patch	multiple cardiac tumors	*TSC2* mutation
		7/20	3/18	66/76				2/7	84/98	1/3
		35.0%	16.7%	86.8%				28.6%	85.7%	33.3%
		SEGA	SEGA	SEGA	–	–	–	–	–	NMI
		1/20	1/18	5/76						0/3
		5.0%	5.6%	6.5%						0.0%
Average age	of first examination performed within the first 16 weeks of life (weeks)	–	1.6 (±3.0)	5.0 (±4.8)	3.7 (±3.9)	9.1 (±6.5)	1.9 (±3.4)	5.3 (±5.3)	1.9 (±3.7)	–
Median age		–	0.4	3.0	2.5	7.0	0.6	2.6	0.3	–
Average age	of pathological lesions onset within the first 16 weeks of age (weeks)	–	1.0 (±1.6)	5.0 (±4.6)	8.6 (±5.9)	11.9 (±7.0)	0.4 (±0.6)	3.8 (±5.4)	2.7 (±4.3)	10.5 (±4.6)
Median age		–	0.1	3.0	8.9	11.9	0.1	1.0	0.8	11.1
Average age	of last examination within the first 16 weeks of life (weeks)	–	2.5 (±3.8)	5.7 (±5.1)	12.0 (±5.3)	9.1 (±6.5)	3.1 (±5.9)	5.9 (±5.4)	4.5 (±4.9)	–
Median age		–	0.7	3.5	14.3	7.0	1.2	4.9	1.7	–

TUS (transfontanelle ultrasonography), abdominal US (abdominal ultrasonography), SE (skin examination), OE (ophthalmological examination), PE (postnatal echocardiography); SEN (subependymal nodules), cortical dysplasia includes cortical tubers and white matter migration lines, SEGA (subependymal giant cell astrocytoma), HMs - hypomelanotic macules, NMI - no mutation identified

### Clinical signs of TSC

Apart from cardiac tumors, the most frequent signs of TSC observed within the first 16 weeks of age were CNS lesions (76/100, 76.0%) (mainly subependymal nodules (71/100, 71.0%) and cortical dysplasia (66/100, 66.0%)), and hypomelanotic macules (35/100, 35.0%). Table 2 summarizes the diagnostic signs that were present in the group of ‘early TSC diagnosis’ in the first 16 weeks of age. Additionally, subependymal giant cell astrocytoma (SEGA), multiple retinal hamartomas, multiple renal cysts and retinal achromic patches were also detected in some children. Nevertheless, none of the patients showed other major (facial angiofibromas, ungual fibromas, shagreen patch, lymphangioleiomyomatosis, renal angiomyolipomas) or minor (dental enamel pits, intraoral fibromas, confetti skin lesions, non-renal hamartomas) diagnostic criteria of TSC.

**Table 2:** TSC signs observed within 16 weeks of age in patients with ‘early TSC diagnosis’ (*n* = 82)

TSC signs	Major criteria						Minor criteria		Pathogenic
	CTs	SEN	CD	SEGA	HMs	MRH	MRC	RAP	TSC1/2
									mutation
No. of patients	82	71	66	5	35	5	8	2	3
Per cent %	100%	86.6%	80.5%	6.1%	42.7%	6.1%	9.8%	2.4%	3.7%

*CTs* cardiac tumors, *SEN* subependymal nodules, *CD* cortical dysplasia: includes cortical tubers and white matter migration lines, *SEGA* subependymal giant cell astrocytoma, *HMs* hypomelanotic macules, *MRH* multiple retinal hamartomas, *MRC* multiple renal cysts, *RAP* retinal achromic patch

Table 3 illustrates the ‘clinical scenarios’, i.e. the association of TSC signs observed within the first 16 weeks of age in the group of ‘early TSC diagnosis’.

**Table 3:** The association of TSC signs within 16 weeks of age in ‘early diagnosed’ children (*n* = 82)

Major criteria				Minor criteria		Genetic diagnosis	
cardiac tumors	At least one CNS lesion (SEN, cortical dysplasia or SEGA)	HMs	multiple retinal hamartomas	multiple renal cysts	retinal achromic patch	TSC1/2 mutation	No. of patients (%)
+	+						37 (45.1%)
+	+	+					24 (29.3%)
+		+					5 (6.1%)
+	+	+		+			3 (3.7%)
+	+		+				3 (3.7%)
+	+			+			3 (3.7%)
+	+					+	2 (2.4%)
+	+	+	+		+		1 (1.2%)
+	+	+		+		+	1 (1.2%)
+	+		+	+			1 (1.2%)
+	+	+			+		1 (1.2%)
+	+				+		1 (1.2%)

*CNS* central nervous system, *SEN* subependymal nodules, cortical dysplasia includes cortical tubers and white matter migration lines, *SEGA* subependymal giant cell astrocytoma, *HMs* hypomelanotic macules

### Genetic testing

Genetic testing was conducted in 49 out of 100 patients (49.0%), including 40 (40/49, 81.6%) patients with ‘early diagnosis of TSC’, 7 (7/49, 14.3%) with ‘late diagnosis of TSC’, and 2 (2/49, 4.1%) with ‘possible TSC’, respectively. A summary of molecular testing results is presented in Table 4. A pathogenic variant was identified in 91.9% of examined children (45/49). In 4 patients (4/49, 8.1%) no causative mutation was detected. However, 2 of those children were diagnosed with TSC based on clinical signs. Other 2 patients with undetected mutation have been included into the group of ‘possible TSC’ as no other signs of the disease were observed. Only in 3 patients (3/49, 6.1%), in whom blood samples were collected for genetic test, the results were obtained before 16 weeks of life. Nevertheless, those children had been already diagnosed with TSC based on clinical signs. However, according to the recent diagnostic criteria [4], the disclosure of a pathogenic variant enabled the ‘late’ diagnosis of TSC in 4 children (4/100, 4.0%) in whom other clinical signs were not present. There was no significant difference between the proportion of *TSC1* and *TSC2* mutations between the group of ‘early’ and ‘late’ TSC diagnosis (*p* > 0,05).

**Table 4:** Results of genetic testing

	Overall	The group of ‘early TSC diagnosis’	The group of ‘late TSC diagnosis’	The group of ‘possible TSC’
No. of patients in whom the genetic tests was conducted	49/100 (49.0%)	40/82 (48.8%)	7/13 (53.8%)	2/5 (40.0%)
*TSC1* mutation	9 (18.4%)	7 (17.5%)	2 (28.6%)	0 (0.0%)
*TSC2* mutation	36 (73.5%)	31 (77.5%)	5 (71.4%)	0 (0.0%)
No mutation identified (NMI)	4 (8.1%)	2 (5.0%)	0 (0.0%)	2 (100%)
Average age of patients when the result was obtained (years)	4.7 ± 7.2	4.3 ± 6.0	3.5 ± 6.4	2.4 ± 0.0
Median age of patients when the result was obtained (years) [range]	1.5	1.4	6.4	2.2
	[0.1–21.0]	[0.1–21.0]	[0.4–18.0]	[2.0–2.4]
No. of mutations detected by age 16 weeks	3/49 (6.1%)	3/40 (7.5%)	0	0
No. of patients in whom genetic testing enabled TSC diagnosis	4/100 (4.0%)	0	4/13 (30.8%)	0

### The group of ‘late diagnosis’ (diagnosis made after age 16 weeks)

In the group of ‘late diagnosis of TSC’ (13 children), the clinical tests performed by age 16 weeks were: transfontanelle ultrasonography in 5 (5/13, 38.5%) and brain MRI in 4 (4/13, 30.8%) cases, abdominal ultrasonography in 7 (7/13, 53.8%) patients, ophthalmological examination in 3 children (3/13, 23.1%), skin examination with Wood’s lamp in 7 cases (7/13, 53.9%), and echocardiography in 13 patients (13/13, 100%). Nevertheless, apart from echocardiography, those tests did not reveal any pathological lesions. In 4 children (4/13, 30.8%) the diagnosis of TSC was made based on the identification of a pathogenic mutation. The average age of the molecular diagnosis in these four patients was 1.3 ± 0.5 years (median age: 1.5 years). Other 9 (9/13, 69.2%) children were eventually diagnosed with TSC based on the presence of cardiac tumors and CNS lesions. The average age of diagnosis in those 9 patients was 2.4 ± 3.1 years (median age: 0.6 years).

### The group of ‘possible TSC’

In the group with not definite TSC diagnosis skin examination and postnatal brain MRI were conducted in all cases. Median age of last follow up was 77 weeks for skin examination and 36.7 weeks for brain MRI. Abdominal ultrasonography or MRI were performed in 3 patients (3/5, 60.0%) with median time of the last examination at age 19.3 weeks. Ophthalmological consultation was done in one child (1/5, 20.0%) at age 17 weeks. Genetic test was performed in two cases (2/5, 40.0%) at a median age of 2.4 years, and no pathogenic variants were identified. Further follow up was not continued, as the patients were under the care of other hospitals and outpatient clinics.

## Discussion

The aim of this study was to define the most useful approach to make the diagnosis of TSC before clinical seizures onset (i.e. before age 4 months) in order to facilitate early EEG monitoring and possible preventative antiepileptogenic treatment intervention. Recent studies showed the beneficial role of this approach in children younger than age 2 years with improvement of cognitive outcome and better seizure control [10, 11]. This strategy is currently applied in the multicenter European Commission project EPISTOP [19]. In the EPISTOP project, patients with the diagnosis of TSC established before seizure onset are monitored with EEG every 4 weeks in the first 6 months of life and every 6 weeks thereafter. The treatment is introduced if ictal discharges in the EEG recording occur, before clinical seizures [19]. Moreover, recent European recommendations on epilepsy treatment in TSC also suggest early introduction of Vigabatrin within 24 months of life if ictal discharges occur, with or without clinical manifestation [18].

However, the early diagnosis of TSC is still a challenge in the majority of cases as diagnostic workup often begins after seizure onset [20]. Therefore, the aim of this study was to define a useful approach that especially the neonatologist and pediatrician can utilize to make the diagnosis of TSC before seizures onset.

In this study, we selected children in whom the first sign of TSC was single or multiple cardiac tumors (rhabdomyomas). Cardiac rhabdomyomas (CRs) are the most frequent cardiac tumors in children and the earliest detectable sign of TSC [1, 21]. They can be diagnosed from the 20th gestational week [21]. Although both single and multiple tumors are associated with TSC [4], the probability of having the disease increases in patients with multiple lesions [22, 23]. Tworetzky et al. diagnosed TSC in 95% of individuals with multiple cardiac tumors compared to 23% with single ones [22]. Conversely, the frequency of CRs in TSC patients varies with age [2, 21, 24]. Overall, about 50% of the patients have cardiac tumors [21]. However, they are seen in higher frequency in children younger than 2 years of age and then they usually regress over time [21].

An additional benefit of early CRs recognition is the possibility to perform antenatal MRI with ultrafast imaging, which, compared to the postnatal study, does not require maternal or fetal sedation [24–27]. Prenatal brain MRI is usually performed in the third trimester, although detection of subependymal nodules (SEN) in the 21st gestational week has also been reported [26]. Moreover, children diagnosed prenatally have a greater chance for improved prognosis as prenatal diagnosis of TSC provides the possibility of personalized pregnancy management and of referring the pregnant woman and child to referenced centers, with a view to early implementing optimal management and EEG monitoring. In our study group prenatal MRI enabled TSC diagnosis in 2/3 of the children (20/30, 66.7%) in whom the test was performed. Nevertheless, one should keep in mind that negative results of antenatal MRI do not exclude TSC. In the remaining 10 children (10/30, 33.3%) with normal prenatal MRI, 8 patients (8/30, 26.7%) were diagnosed with TSC after birth due to the disclosure of CNS lesions in postnatal scans (6 children) or identification of a pathogenic mutation (2 children). Therefore, postnatal brain MRI should be performed even when antenatal MRI does not show abnormalities. As a matter of fact, postnatal brain MRI was performed within age 16 weeks in the vast majority of the children in our cohort (83/100, 83.0%), and it significantly contributed to early TSC diagnosis (Table 1 and Table 3).

Other major diagnostic criteria of TSC are dermatological findings, with hypomelanotic macules (HMs) being a major diagnostic criterion of TSC and the most common dermatological manifestation, present in 90–98% of patients [1, 3, 4]. Furthermore, as other skin signs usually appear in older children or adults, HMs are the earliest detectable skin manifestation and might be seen even in newborns [1–3, 28]. In our cohort, at least 3 HMs were found early in 1/3 of the patients (35/100, 35.0%) (Table 1). Moreover, in 5 children (5/82, 6.1%), in the group of ‘early TSC diagnosis’, they considerably contributed to the diagnosis when other extra-cardiac manifestations were not present (Table 3). Therefore, although HMs are not pathognomonic for TSC and may be observed in other conditions as well as in healthy individuals [28], they facilitate the early diagnosis of the disease.

Detection of ophthalmological changes may also aid the TSC diagnosis. Diagnostic ophthalmological findings comprise multiple retinal hamartomas as a major criterion and multiple achromic patches as minor ones [4]. They have been reported in 39–50% of TSC patients and may be found at any age [1, 29, 30]. In the group of ‘early TSC diagnosis’ we found multiple retinal hamartomas in 5 (5/82, 6.1%) and achromic patches in 2 (2/82, 2.4%) patients. However, these signs were not crucial for the diagnosis in any case, as other diagnostic criteria (CRs, CNS lesions or HMs) had been detected earlier.

Renal manifestations of TSC may also contribute to the diagnosis. They include renal angiomyolipomas (AMLs), renal cysts and renal carcinoma. The first two are major and minor diagnostic criteria, respectively [4]. AMLs are present in as many as 80% of patients, but they usually develop after the third year of age [1–3]. Accordingly, we have not found AMLs in any of our patients in the first 16 weeks of age. On the other hand, multiple renal cysts may be present since infancy [1–3]. Moreover, autosomal dominant polycystic kidney disease (ADPKD) may coexist with TSC as large deletions of *TSC2* may also involve *PDK1*, whose mutations are responsible for ADPKD [31]. In our cohort, we found renal cysts in only 8 patients (8/100, 8.0%). However, as multiple renal cysts are a minor diagnostic criterion, in terms of the absence of other minor signs among those patients, renal cysts were only additional features and did not decide about the diagnosis.

Since 2012 the identification of a pathogenic mutation in *TSC1* or *TSC2* is sufficient for establishing the diagnosis [4]. Therefore, DNA testing became a useful diagnostic tool. A pathogenic mutation is reported to be found in 75–90% of TSC patients [32]. This percentage is considerably increased when full gene coverage and next generation sequencing (NGS) are used [32]. Genetic testing results were not obtained early in our study in the vast majority of patients (46/49, 93.9%) in whom DNA testing was performed. Nevertheless, the availability of molecular testing is improving, and genetic analyses may significantly contribute to early TSC diagnosis in the future. In our study identification of the pathogenic mutation enabled the ‘late’ diagnosis in the absence of other clinical signs in 4 children (4/100, 4.0%).

Our study shows that early diagnosis of TSC, before clinical seizure onset, is feasible. In the vast majority of our patients (82/100, 82.0%) the diagnosis was established before the end of the 4th month of life (16 weeks), which is regarded as the usual time of clinical seizure onset. Thus in this study the cut-off point between the groups was 16 weeks (4 months), it need to be acknowledged that epileptogenic process begins even earlier in TSC patients and clinical seizures are preceded by EEG abnormalities [8, 10–13]. In our recent study we reported that regular surveillance EEG and early implementation of the antiepileptic treatment when paroxymal discharges on EEG record occur but before clinical seizures was beneficial for developmental outcome and seizure control [10].

Moreover, Chung et al. reported that the group of TSC patients diagnosed prior to seizures had lower percentage of refractory seizures and lower number of trailed antiepileptic drugs despite the fact that none of those children had a regular surveillance EEG as in our previous study [33]. Furthermore, the prevalence of severe developmental disability was also significantly lower [33]. The explanation of those data is that considering the awareness of the association between seizures and developmental outcome parents were probably earlier educated about seizures recognition and clinicians vigilance was also greater in patients diagnosed before seizures [33]. Consequently,seizures had been noticed and antiepileptic drugs had been implemented earlier, which increased a chance for better seizure control and improved the clinical outcome [10, 12, 13, 17, 33].

Therefore, as children diagnosed with TSC before seizure onset have better prognosis [10, 13, 33] it needs to be emphasized that early TSC diagnosis is becoming pivotal and should be established as soon as possible.

Although our study group represents less than a half of TSC population from our clinics born between 1990 and 2016 (20% from the Department of Neurology and Epileptology of The Children’s Memorial Health Institute of Warsaw and 13% from the Child Neuropsychiatry Unit and Epilepsy Center of the San Paolo Hospital in Milan), we observed that the number of children referred before 4 months of life significantly increased in recent years. It is probably due to the increased awareness of obstetricians, neonatologists and pediatricians. In the EPISTOP study we are collaborating with pediatricians, child cardiologist, neonatologist and obstetrician from different medical centers and emphasizing the importance of early recognition of TSC signs and referral children for further diagnosis.

**Fig. 1:**
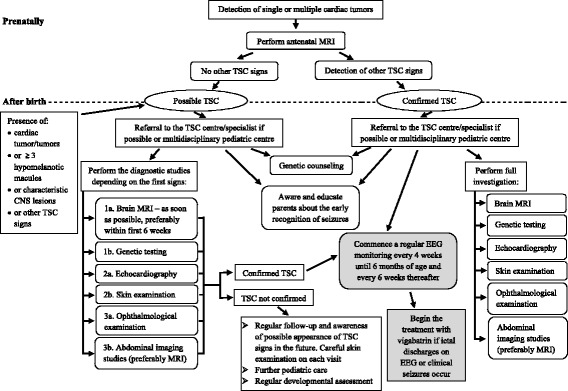
Diagnostic algorithm for early TSC diagnosis and management in infants. In patients suspected for TSC the diagnostic studies should be performed early, as soon as possible. Ordinal numbers in diagnostic studies for patients with possible TSC indicate the importance and priority of those studies for early TSC diagnosis. However, it is recommended to perform all the tests

In Fig. 1 we propose the diagnostic and management algorithm for early TSC diagnosis. Especially obstetricians, neonatologists and pediatricians could benefit from this flowchart, as they are usually the first physicians who take care of newborns and infants with suspected TSC.

If TSC is suspected prenatally, mother should be referred to maternal fetal medicine to perform fetal brain MRI. If the TSC diagnosis is prenatally confirmed, child should be referred preferably to TSC centre/specialist just after birth and all recommend studies should be conducted [34]. If the TSC centre/specialist is not available, the patient should be under the care of multidisciplinary pediatric centre. Moreover, regular EEG monitoring every 4 weeks needs to be commence since birth. Parents should be aware and educated about the risk and recognition of seizures and genetic counseling need to be provided.

If TSC is not confirmed prenatally or a child is suspected of having TSC after birth, all diagnostic tests should be performed as soon as possible preferably in TSC centre. A brain MRI is one of the most useful tests in early TSC diagnosis, detecting pathological lesions in the vast majority of patients, and should be performed preferably within the first 6 weeks of life. Although the turnaround time of genetic analysis can be long in some countries, DNA testing is a useful tool to confirm the diagnosis of TSC and should be also requested as soon as possible, especially in the absence of sufficient clinical signs. Due to the design of the study all our patients had cardiac tumors. However, as CRs are a frequent sign of TSC, especially in infants, echocardiography may also facilitate the diagnosis in patients suspected of the disease and should be early performed. Skin examination with Wood’s lamp is helpful in identifying hypomelanotic macules. It is also an easy and cheap test, and should be performed in every patient during each visit. We also recommend an early ophthalmological consultation and abdominal ultrasonography or MRI in patients suspected of having TSC in the absence of other signs or when brain MRI cannot be performed early.

### Limitations of the study

Some limitations of the study should be acknowledged. It is a retrospective study and we did not analyze the data of seizure onset and developmental outcome in our cohort. However, we provide the literature data about the improved prognosis of children diagnosed prior to seizures. Moreover, we included in this study only children in whom the first sign of the disease was cardiac tumor/s. However, early diagnosis of TSC could also be made in patients without cardiac tumors, although it might be more challenging. Nevertheless, in recent years most children have been referred to our clinics in infancy with suspected TSC based on the presence of single or multiple cardiac tumors. Therefore, we believe that this study will contribute to the earlier diagnosis of TSC, allowing the early introduction of EEG monitoring and antiepileptic treatment with a view to the improvement of neurodevelopmental outcome.

## Conclusions

Early diagnosis of TSC before seizure onset is feasible, and - in the light of recent studies and recommendations [10, 11, 13, 17–19, 33] - it is becoming pivotal for epilepsy management with possible improvement of the clinical outcome. Early TSC diagnosis is mostly based on clinical signs. Brain MRI, echocardiography and careful skin examination with Wood’s lamp should be performed early in every young patient suspected of having TSC. Genetic testing for *TSC1/2* mutations should also be conducted early, when possible. However, as in most cases the turnaround time is long and in some patients the mutation is not found, clinical studies for TSC signs remain the most substantial for early TSC diagnosis and cannot be abandoned.

## Abbreviations

ADPKD: Autosomal dominant polycystic kidney disease
AMLs: Angiomyolipoma
CD: Cortical dysplasia
CNS: Central nervous system
CRs: Cardiac rhabdomyoma
CTs: Cardiac tumors
EEG: Electroencephalography
HMs: Hypomelanotic macules
MRC: Multiple renal cysts
MRH: Multiple retinal hamartomas
MRI: Magnetic resonance imaging
mTOR: Mammalian target of rapamycin
NGS: Next generation sequencing
NMI: No mutation identified.
OE: Ophthalmological examination
PE: Postnatal echocardiography
RAP: Retinal achromic patch
SE: skin examination
SEGA: Subependymal giant cell astrocytoma
SEN: Subependymal nodules
TSC: Tuberous sclerosis complex
TUS: Transfontanelle ultrasonography
US: Abdominal ultrasonography
